# Correlation of Medical Comorbidities and Upper Airway Measurements among Dental Patients at Risk of Developing Obstructive Sleep Apnea

**DOI:** 10.3390/clinpract12030034

**Published:** 2022-05-06

**Authors:** Kar Yi Lin, Pei Ying Eow, Shivani Kohli, Swarna Yerebairapura Math

**Affiliations:** School of Dentistry, International Medical University, Bukit Jalil, Kuala Lumpur 57000, Malaysia; lin.karyi@student.imu.edu.my (K.Y.L.); eow.peiying@student.imu.edu.my (P.Y.E.); swarnayerebairapura@imu.edu.my (S.Y.M.)

**Keywords:** Obstructive Sleep Apnea, cone beam computed tomography, medical comorbidities, OSA symptoms, upper airway dimensions

## Abstract

Obstructive Sleep Apnea (OSA) is a partial or total upper airway collapse resulting in sleep-breathing disturbances. There are many medical comorbidities associated with OSA; hence, this study is important as the prevalence of patients with medical comorbidities associated with OSA is increasing. The study aimed to correlate medical comorbidities and OSA symptoms of the patients along with their upper airway dimensions using Cone Beam Computed Tomography (CBCT) scans to identify patients at risk of developing OSA. This cross-sectional study included patients who had CBCT imaging taken between 2014 and 2020. A questionnaire was used to gather information on patients’ medical history and OSA symptoms. The upper airway dimensions of the CBCT scans were evaluated before logistic regression and Fisher’s exact test were carried out to determine the relationships between the variables. *p* ≤ 0.05 was considered statistically significant. Logistic regression revealed an association of longer length (*p* = 0.016), smaller total volume (*p* = 0.017) and width (*p* = 0.010) of upper airways with hypertension. Furthermore, loud snoring was seen in patients with hypertension, heart disease and obesity whereas difficulty concentrating during the day was present in subjects with deviated nasal septum, tonsillitis and depression. For upper airway dimensions, a smaller average volume was associated with loud snoring (*p* = 0.037), difficulty concentrating during the day (*p* = 0.002) and mood changes (*p* = 0.036). A larger anterior-posterior dimension was also associated with excessive daytime sleepiness (*p* = 0.042), difficulty concentrating during the day (*p* < 0.001) and mood changes (*p* = 0.009). Longer airway length was additionally found to be associated with loud snoring (*p* = 0.021). CBCT taken for dental investigations could be correlated with patients’ medical history and OSA symptoms to screen patients at risk of OSA.

## 1. Introduction

Obstructive Sleep Apnea (OSA) is a medical condition of increasing prevalence where there is partial or complete collapse of the airway which transports oxygen to our body [1,2]. This condition, which obstructs the upper airway, can cause a decrease in oxygen saturation in our body and may even cause abrupt arousals during sleep [3]. This may also lead to loud snoring or even cause breathing to stop momentarily [1]. According to the University of Toronto, more than 80% of OSA patients remain undiagnosed due to the lack of awareness of this condition [4].

There are many causes that can be associated with the occurrence of Obstructive Sleep Apnea. According to a literature review, the main cause of this condition is the weakening of the genioglossal muscle, leading to a reduction in the pharyngeal dilator muscles which expand the pharynx [5]. Other causes of this condition include additional tissues of the soft palate, hypertrophy of the tonsils or even an abnormal shape and size of the tongue. Macroglossia has been found to be one of the many causes of developing OSA [5]. In addition, patients who have certain skeletal abnormalities or changes in the soft tissue structures of the neck, most commonly found in obese patients, who also have increased amount of adipose tissue around the neck, may have an altered anatomy of the upper airway, predisposing them to OSA [5,6]. Other risk factors of OSA are the male gender, frequent use of alcohol, smoking and habitual snoring [1,7]. 

Patients who have OSA may complain of excessive sleepiness during the day, unrefreshing sleep, daytime fatigue and a lowered concentration span [3]. These can lead to behavioural irritability and may affect their memory. Night-time symptoms can include loud snoring, sudden awakening due to lack of oxygen and waking up with symptoms of dry mouth. Motor vehicle-related accidents have been reported by some due to the extreme drowsiness felt [3]. In addition to this, there are also many long-term effects which can affect individuals with OSA, such as increased heart rate, chronic increase in blood pressure, increased risk of heart-related diseases and stroke, decreased glucose tolerance and impaired insulin resistance [8]. 

Furthermore, OSA has also been found to be associated with other medical comorbidities such as cardiovascular and metabolic diseases [9]. Many studies have found strong evidence indicating the relationship between OSA and hypertension. Researchers believe this may be due to constant sleep disturbance and intermittent drops in oxygen levels, leading to chronic systemic hypertension or even arrhythmias, which may result in higher morbidity and mortality rates in these patients [4,7]. In addition, untreated OSA in diabetic patients may increase risk of neuropathy, diabetic retinopathy and diabetic nephropathy [9]. Incidence of Gastroesophageal Reflux Disease (GERD) was also found to be significantly elevated in patients with OSA. OSA has also been found to be associated with other conditions, such as COPD and asthma, which may cause reduction in overall quality of life [8]. 

As mentioned previously, OSA may become a dangerous condition if not diagnosed and managed in the early stages. Therefore, it is necessary to attempt to diagnose all patients with OSA to provide early intervention [4]. Dental professionals play an important role in being one of the first individuals in identifying patients with OSA [10]. A routine dental examination of patients may reveal risk factors for developing OSA and smaller upper airway dimensions on CT scans can guide provisional diagnosis for earlier referral. A simple screening tool can then be used to determine whether the patient is in fact at risk of OSA before further medical intervention can be carried out [10]. As this condition is becoming more prevalent, there may be many patients with undiagnosed OSA going to a dental clinic for regular dental treatment [11]. Therefore, it is advised that all dental healthcare professionals be educated and trained appropriately [12]. 

This study aims to evaluate the association between patients’ medical comorbidities, OSA symptoms and CBCT findings of upper airway dimensions among patients at risk of OSA. This study proves to be important as there is an increasing prevalence of patients with medical comorbidities associated with OSA [7]. 

## 2. Materials and Methods

Ethical approval was obtained from the IMU Research Review Committee prior to the commencement of this research. This cross-sectional study was carried out to evaluate the association between Cone Beam Computed Tomography (CBCT) findings of the upper airway dimensions and medical comorbidities among patients suspected with OSA. This study was carried out based on interpretation of patient’s CBCT scans from May 2014 to January 2020 in the Oral Health Centre of International Medical University (OHC, IMU). Inclusion criteria for this study were patients aged 18 years and above who have undergone CBCT scans for any past dental treatment in the Oral Health Centre (OHC, IMU). The size of the field of view of the CBCTs included in this study involved the nasopharynx and oropharynx region with proper tongue positioning. Exclusion criteria were edentulous patients, improper CBCT imaging displaying a posterior positioning of the tongue which may affect measurements and significant mandibular retrognathia or prognathia.

**Figure 1:**
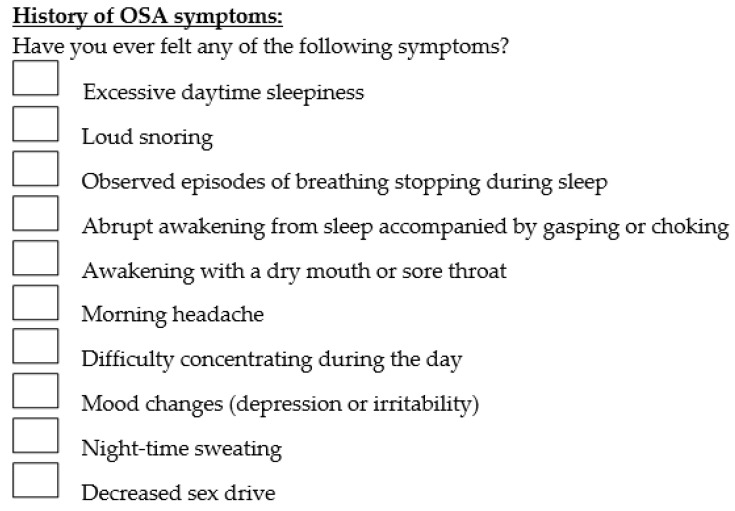
Questions regarding history of any OSA symptoms.

**Figure 2:**
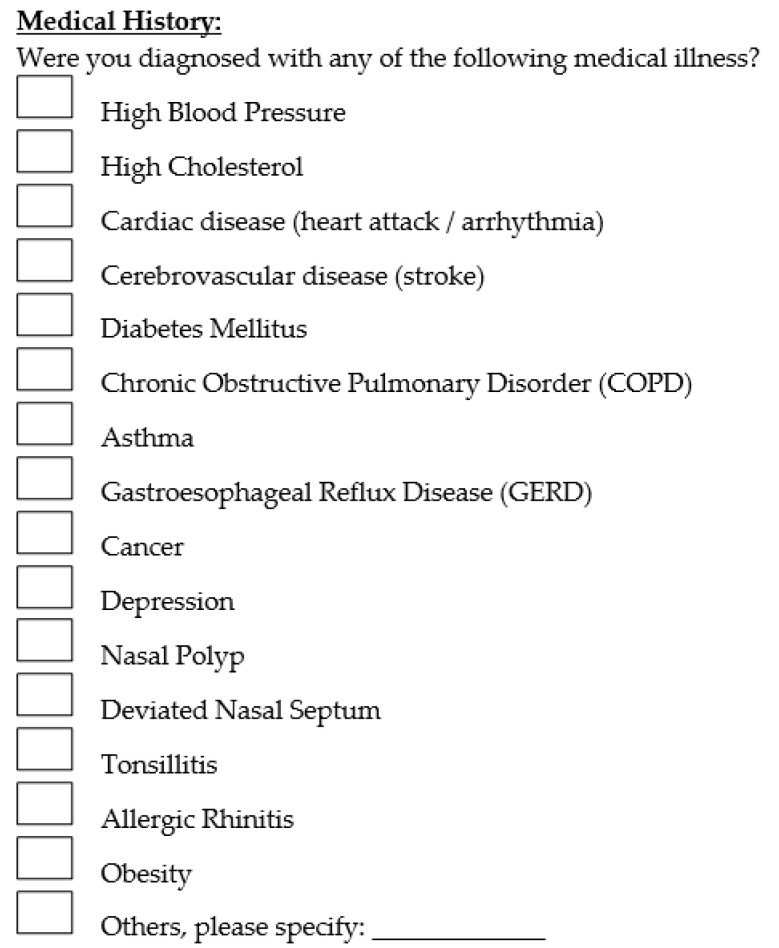
Questions regarding past medical history.

Questions regarding history of OSA symptoms included the presence of excessive daytime sleepiness, loud snoring, morning headache, etc. The medical illnesses included in the questionnaire were hypertension, hypercholesterolemia, cardiac disease, cerebrovascular disease, diabetes mellitus, Chronic Obstructive Pulmonary Disorder (COPD), asthma, Gastroesophageal Reflux Disease (GERD), cancer, depression, nasal polyp, deviated nasal septum, tonsillitis, allergic rhinitis and obesity. Patients were also asked to state whether they are currently taking any medications or supplements. Presence of any pre-existing OSA symptoms and comorbidity diagnoses were solely based on the responses obtained from the questionnaire. The questionnaire used is shown in Figure 1 and Figure 2 below.

1269 CBCT scans were obtained and these patients were contacted by e-mail. All patients were required to fill out an online questionnaire regarding the presence of any positive medical comorbidities and OSA-related symptoms. Along with this questionnaire, a consent form and a study information sheet were sent to the patients to obtain informed consent of their willingness to participate in this research study.

**Figure 3:**
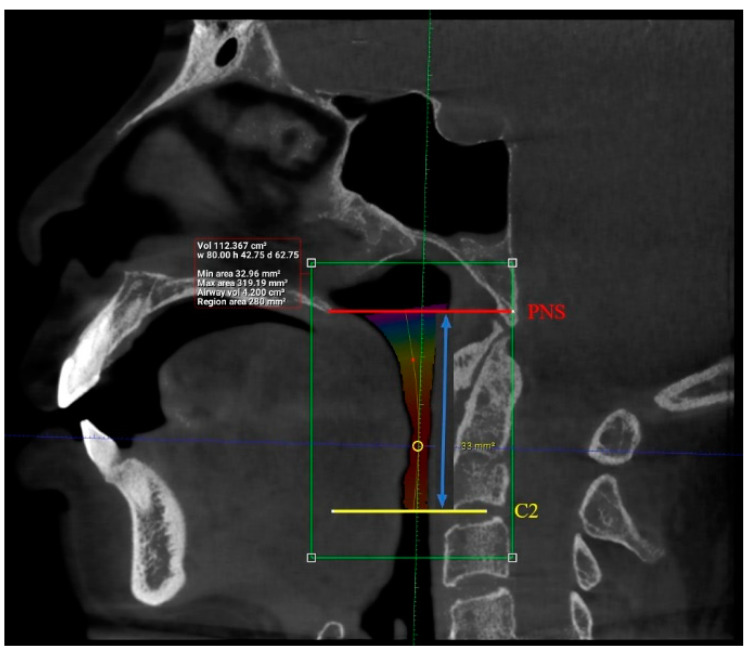
Sagittal view of the oropharynx region, isolating the region from the posterior nasal spine (PNS) to cervical vertebrae C2. The total length, total airway volume of the oropharynx and average airway volume of the oropharynx were measured. Yellow circle represents the narrowest portion of the upper airway.

**Figure 4:**
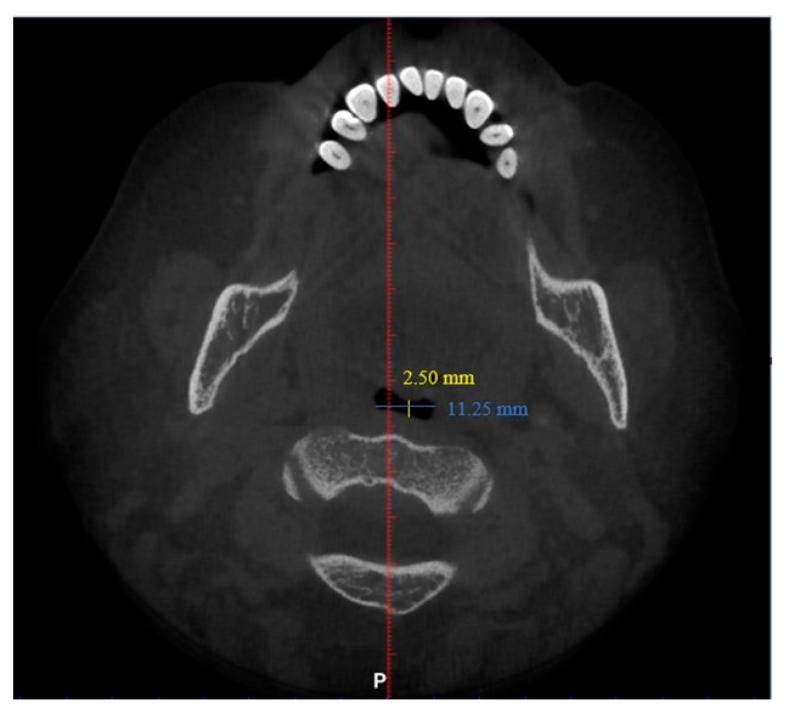
Measurement of anterior-posterior dimension (yellow) and width (blue) of the narrowest axial slice of the upper airway.

Interpretation of patients’ CBCT scans in OHC, IMU were carried out after they agreed to participate in the study and completed the online questionnaire. The upper airway dimensions in the CBCT scans were measured using Planmeca Romexis software. The oropharynx region beginning from the posterior nasal spine to cervical vertebrae C2 were isolated from the data and analyzed, as seen in Figure 3. CBCT findings that were computed from the sagittal view included the length of the upper airway, total airway volume of the oropharynx and the average airway volume of the oropharynx. The anterior-posterior measurement and width of the upper airway were then measured using the narrowest portion of the upper airway in the axial view, as demonstrated in Figure 4.

Inter-examiner consistency was assessed using Intraclass Correlation (ICC) and demonstrated good to excellent reliability.

Among all the patients contacted, 101 patients agreed to participate in this research study, thereby achieving our targeted sample size of 100 patients. The targeted sample size was calculated based on Cochran’s sample size formula with Z value set at 1.96 for the confidence level of 95%, d = 0.05 and prevalence of 7% [13,14,15].

For data analysis, all the data were tabulated and analyzed using SPSS software version 22.0. Frequency distributions were determined for the demographic data, medical comorbidities and OSA symptoms. Descriptive statistics were then determined for the upper airway dimension parameters. A logistic regression test was carried out to predict the relationship between medical comorbidities and upper airway dimensions, as well as OSA symptoms and upper airway dimensions. Fisher’s exact test was also conducted to determine whether there was a significant relationship between medical comorbidities and OSA-related symptoms. *p* ≤ 0.05 was considered statistically significant for all tests.

## 3. Results

101 patients were included in the study, 46 males and 55 females, ranging from 18 to 77 years. Table 1 shows the frequency distribution of gender, age, race, medical comorbidities and OSA symptoms of the patients involved in this study.

The most common medical comorbidities of the patients involved in this study were hypertension and high cholesterol. Meanwhile, the majority of the patients involved experienced loud snoring, while other common symptoms included excessive sleepiness during the day and awakening often with dry mouth or sore throat.

After compiling the frequency distribution of each medical comorbidity and OSA symptom, all the data were then cross tabulated, bearing in mind that some patients diagnosed with a particular medical condition may have one or more OSA symptom. For example, out of all the people with hypertension, 9 (56.3%) had excessive daytime sleepiness, 7 (43.8%) experienced loud snoring, 1 (6.3%) respondent each experienced stopped breathing during sleep and was abruptly awakened from sleep due to gasping or choking, 5 (31.3%) awakened with dry mouth or sore throat in the morning, 2 (12.5%) each had morning headaches, experienced difficulty concentrating during the day and mood changes, and lastly 1 (6.3%) respondent each experienced night sweats and decreased sex drive. Table 2 shows the frequency distribution of those with medical comorbidities and history of OSA symptoms.

**Figure 5:**
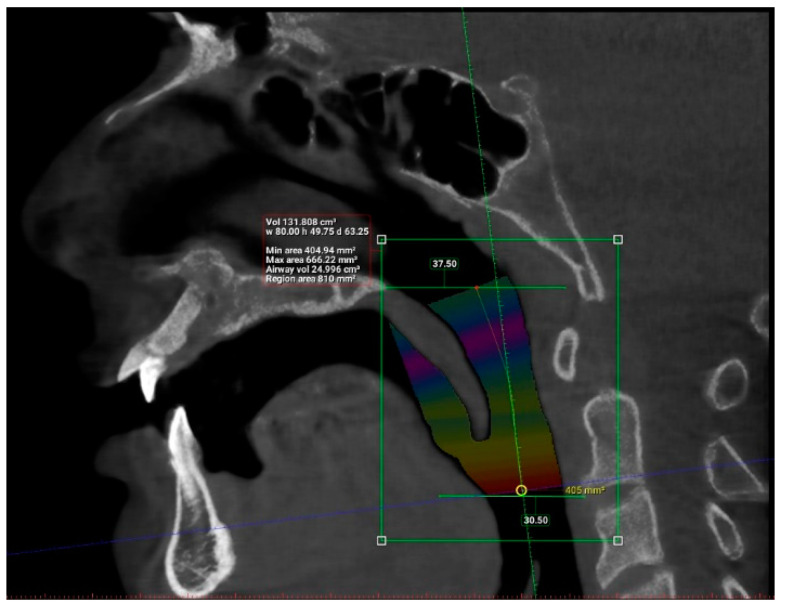
Sagittal view of the upper airway of a patient without hypertension.

**Figure 6:**
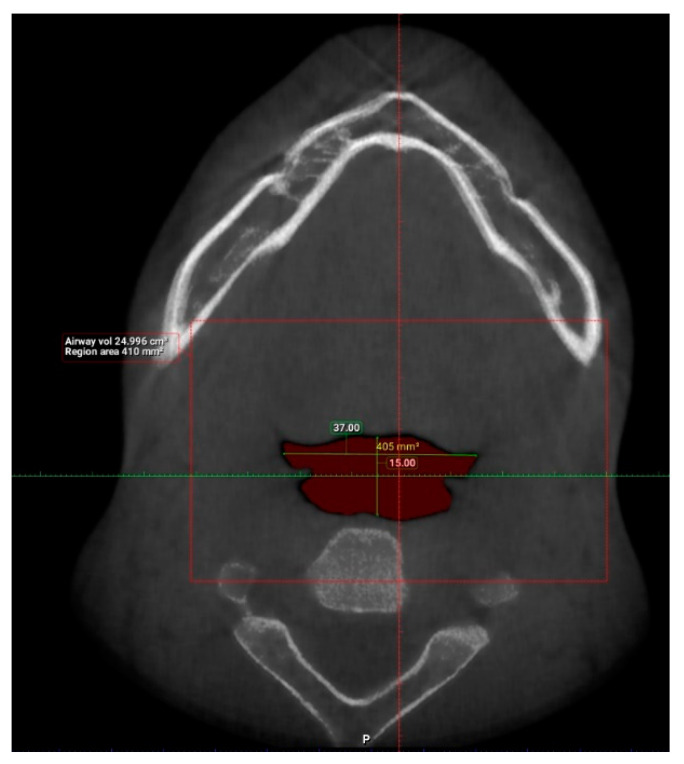
Narrowest axial portion of the upper airway of a patient without hypertension.

**Figure 7:**
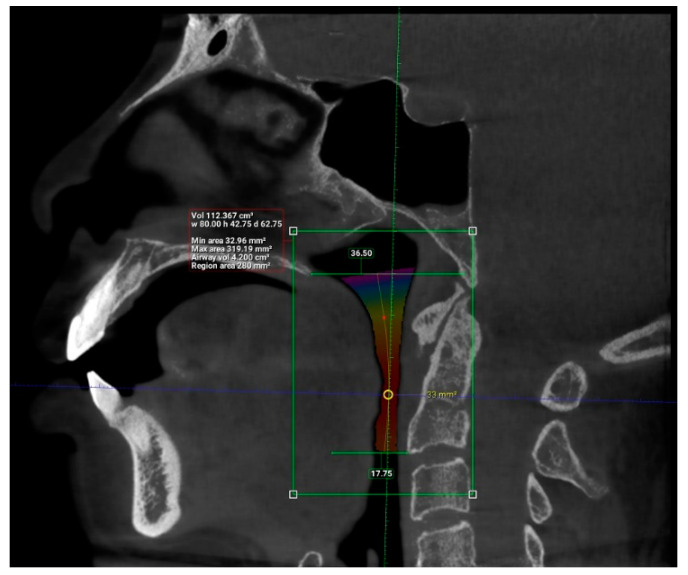
Sagittal view of the upper airway in a hypertensive patient.

**Figure 8:**
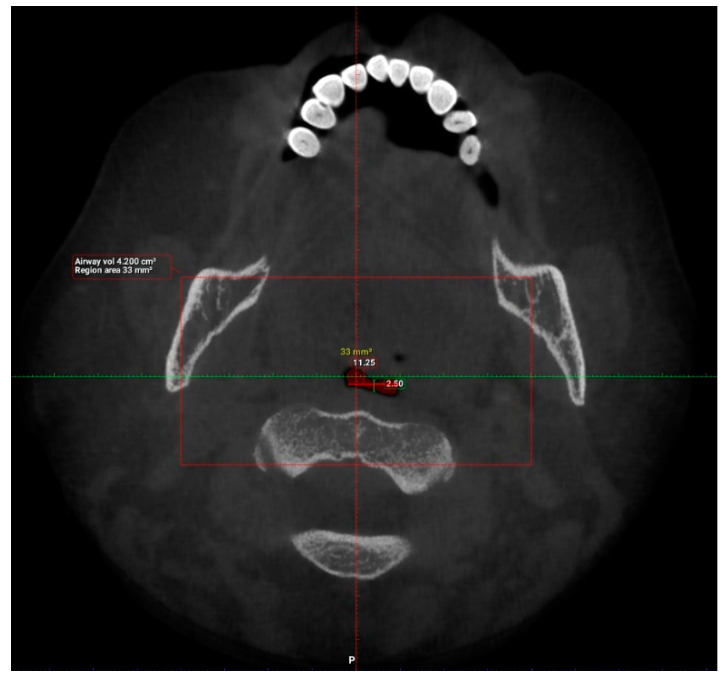
Narrowest axial portion of the upper airway in a hypertensive patient.

The upper airway of each patient was segmented and different parameters were measured in the sagittal and axial view, as demonstrated in Figure 5 and Figure 6 (patient without hypertension) and Figure 7 and Figure 8 (hypertensive patient).

The descriptive statistics for each of the parameters measured is displayed in Table 3.

Logistic regression analysis was carried out to predict the relationship between medical comorbidities and upper airway dimensions (Table 4) as well as OSA symptoms and upper airway dimensions (Table 5). From the results obtained, length (OR = 1.341; 95% CI: 1.056–1.705, *p* = 0.016) and total volume (OR = 0.896; 95% CI: 0.819–0.980, *p* = 0.017) of upper airway as well as width of the narrowest portion of the upper airway (OR = 0.781; 95% CI: 0.648–0.942, *p* = 0.010) were found to be associated with hypertension. 

In addition to that, anterior-posterior dimension of the narrowest portion of the upper airway was found to be associated with excessive daytime sleepiness (OR = 1.243; 95% CI: 1.008–1.533, *p* = 0.042) while length (OR = 1.154; 95% CI: 1.022–1.304, *p* = 0.021) and average volume of upper airway (OR = 0.711; 95% CI: 0.515–0.980, *p* = 0.037) were associated with loud snoring in these patients. In addition, average volume of the upper airway (OR = 0.471; 95% CI: 0.291–0.765, *p* = 0.002) and anterior-posterior dimensions of the narrowest portion of the upper airway (OR = 2.097; 95% CI: 1.358–3.238, *p* < 0.001) were found to be associated with difficulty concentrating during the day, with high significance values. Similar parameters, average volume of the upper airway (OR = 0.729; 95% CI: 0.542–0.980, *p* = 0.036) and anterior-posterior dimensions of the narrowest portion of the upper airway (OR = 1.477; 95% CI: 1.103–1.979, *p* = 0.009), were found to be associated with mood changes.

Fisher’s exact test (Table 6) was then carried out to determine whether there was a significant association between all the medical comorbidities (hypertension, high cholesterol, heart disease, diabetes, asthma, GERD, cancer, depression, deviated nasal septum, tonsillitis, allergic rhinitis and obesity) and OSA symptoms. From the results obtained, it was found that hypertension (*p* = 0.016) and heart disease (*p* = 0.038) have significant associations with loud snoring. Depression had a significant relationship with awakening with dry mouth or sore throat (*p* = 0.033), concentration difficulties during the day (*p* = 0.050) and mood changes (*p* = 0.005). A deviated nasal septum was found to have a significant association with awakening with dry mouth or sore throat (*p* = 0.033), morning headache (*p* = 0.001) and difficulty concentrating during the day (*p* = 0.050). Tonsillitis was also found to have a significant association with morning headaches (*p* = 0.021) and difficulty concentrating during the day (*p* = 0.034). Lastly, obesity also had a significant relationship with loud snoring (*p* = 0.014).

## 4. Discussion

OSA has been found to be associated with certain medical comorbidities and its prevalence has been increasing in recent years. This study is important in correlating the association between medical comorbidities, OSA symptoms and upper airway dimensions, as seen in CBCT scans among dental patients at risk of developing OSA. According to the University of Toronto, OSA can be associated with several medical comorbidities such as cardiovascular disease, cerebrovascular disease, GERD, metabolic diseases and obesity [16]. Symptoms of OSA can range from daytime sleepiness, behavioural irritability such as depression, sexual dysfunction, heavy snoring, sudden awakening with noisy breathing and dry mouth [17]. OSA patients were also found to have narrower upper airway width, smaller anterior-posterior distance, smaller average volume and total volume, and larger airway length [18].

Results from our study indicated a significant relationship between hypertension and length of upper airway, total volume of upper airway and width of the narrowest portion of the upper airway. Results reveal that with every one unit increase in the length of the upper airway, the odds of being diagnosed with hypertension is 1.341. In addition, a one unit increase in total volume of airway or width will result in lowered odds of having hypertension by 0.896 and 0.781, respectively. From these results, an association can be established between hypertension and larger upper airway length, smaller total volume of airway and smaller width of upper airway, indicating an increased risk of developing OSA. It is important to note that, although these variables have associations with one another, the exact cause-and-effect relationship is not identified in this study. Results from our study proved the claim by Worsnop and colleagues which demonstrated an association between hypertension and OSA [19,20]. A study which used MRI to determine airway diameter also confirmed that patients with OSA had smaller upper airway width and concluded that this parameter can be used as an independent predictor of OSA [21]. This claim is also supported by Enciso and colleagues which determined that patients with narrow upper airway width were 3.9 times more likely to be diagnosed with OSA [22]. 

Several medical comorbidities were also found to have significant relationships with OSA symptoms. Hypertension, heart disease and obesity have significant effects on loud snoring, indicating that these patients may be at risk of developing OSA. A study by Silverberg stated that OSA is a medical condition that is commonly associated with hypertension and cardiovascular diseases [23]. Associations between heart disease and OSA have also been evident in middle aged men [24]. Clinical studies conducted by other researchers such as Gami and colleagues have revealed that OSA and obesity have a two-way relationship, such that obesity may predispose patients to OSA and OSA may itself lead to obesity in some patients, due to its symptoms of sleep deprivation and affected metabolism [25,26]. Other studies also found significant associations between obesity and OSA [26,27]. 

In addition, results from our study also indicate significant associations between loud snoring and upper airway length, as well as average volume. A one unit increase in upper airway length led to increased odds of loud snoring by 1.154. On the other hand, a one unit increase in average airway volume resulted in decreased odds of loud snoring by 0.711. This indicates that patients with loud snoring tend to have longer upper airway length and lower average airway volume, which puts them at increased risk of being diagnosed with OSA [18]. The results from our study supported the claim by Lugaresi that supine position during sleep may cause snoring and mild OSA in patients who have a history of heavy snoring. He claimed that snoring and OSA are two aspects of the same disorder, which may be worsened by abnormally narrow airways [28]. In a study by Woodhead and colleagues, it was also noted that 46% of participants who presented with snoring in their study had a high incidence of OSA [29].

Of the patients with deviated nasal septum, tonsillitis and depression in this study, 50% or more also had significant associations with daytime concentration difficulty, one of the symptoms of OSA, while 100% of the patients with depression also had significant mood changes. Deviated nasal septum and tonsillitis have been found to be related to OSA due to restricted nasal airflow and increased resistance to airflow [30,31]. Mood changes like irritability, depression and anxiety have also been identified in patients who have untreated OSA by previous researchers, which support the results from our study [7,32,33,34]. In addition, daytime concentration difficulty and mood changes also had significant associations with average airway volume and anterior-posterior dimensions of the upper airway. A one unit increase in average airway volume resulted in decreased odds of having concentration difficulty during the day and mood changes by 0.471 and 0.729, respectively; while for every one unit increase in anterior-posterior airway dimensions, odds of having difficulty concentrating during the day and mood changes increased by 2.097 and 1.477, respectively. In other words, patients who have difficulty concentrating during the day or frequent mood changes usually have a smaller average airway volume and bigger anterior-posterior airway dimensions. Smaller average airway volume is one of the indicators of OSA, as stated in a study by Buchanan, although anterior-posterior dimensions tended to be smaller in OSA patients, which differed from our study [18]. 

Previous clinical studies have also indicated that OSA may be associated with dyslipidemia such as high total cholesterol, high low density lipoprotein (LDL), high triglycerides (TG) and low high density lipoprotein (HDL) [35,36,37,38,39]. However, similar results were not reflected in our study as the analysis showed no significant relationship between high cholesterol and upper airway parameters or OSA symptoms. In a systematic review on common comorbidities associated with OSA, it was found that cancer, GERD and respiratory illnesses such as asthma were associated with OSA. They concluded that these medical comorbidities were frequently found in patients with OSA and appeared to be a potential trigger for poorer prognosis as it may cause chronic organ damage [7,9,39]. Several researchers have evaluated the possible association between allergic rhinitis and OSA and have found both conditions to be closely related due to reduced airway diameter in these patients [40,41,42]. In our study, only ≤10% of the patients had each of the medical condition stated above; hence, the results obtained were not significant as the sample size of each of these medical comorbidities was too small.

There are limitations to this study. The first is due to a modest sample size of patients and, secondly, lack of verification on the information obtained. The patients were selected based on their responses to the questionnaire regarding presence of any positive medical history and this information was not verified with a medical report from each patient. Another limitation was that intra-oral clinical examination was not conducted for these patients in the study. Better identification of risk of developing OSA could have been achieved if clinical findings and CBCT images were correlated with the patients’ medical histories. It is also important to note that this study demonstrated an association between certain medical comorbidities and OSA symptoms with patients at risk of being diagnosed with OSA. However, these patients should be referred to a medical practitioner for polysomnography (PSG) to be properly diagnosed.

## 5. Conclusions

Much research has been conducted on the relationship between medical comorbidities and risk of developing OSA as the prevalence of patients with OSA increases worldwide. This study demonstrates that hypertensive patients tend to have larger upper airway length, smaller total airway volume and smaller width of upper airway, predisposing them to the risk of developing OSA. Patients with hypertension, heart disease, obesity, deviated nasal septum, tonsillitis and depression experienced OSA symptoms, which were also significant with respect to increased upper airway length and lower average airway volume, increasing the risk of being diagnosed with OSA.

As the prevalence of OSA is increasing, dental practitioners should be equipped with basic knowledge about OSA, including its clinical features and related medical conditions in order to identify patients at risk of developing OSA. From the results obtained through our study, CBCT imaging taken for routine dental treatment, along with patients’ medical comorbidities and OSA symptoms proved to be useful in screening dental patients at risk of developing OSA; thus, early intervention can be provided to such patients. 

## Figures and Tables

**Table 1:** Frequency distribution of gender, age, race of the patients, medical comorbidities and OSA symptoms.

Factor	Frequency	Percentage (%)
**Gender**		
Male	46	45.5
Female	55	54.5
**Age**		
18–27 years old	33	32.7
28–37 years old	24	23.8
38–47 years old	11	10.9
48–57 years old	11	10.9
58–67 years old	16	15.8
68–77 years old	6	5.9
**Race**		
Malay	11	10.9
Chinese	76	75.2
Indian	9	8.9
Others	5	5.0
**Medical Comorbidities**		
Hypertension	16	15.8
High Cholesterol	10	9.9
Heart Disease	2	2.0
Diabetes	6	5.9
Asthma	6	5.9
GERD	2	2.0
Cancer	4	4.0
Depression	3	3.0
Deviated Nasal Septum	3	3.0
Tonsillitis	6	5.9
Allergic Rhinitis	9	8.9
Obesity	9	8.9
**OSA Symptoms**		
Excessive Daytime Sleepiness	37	36.6
Loud Snoring	57	56.4
Stopped Breathing During Sleep	4	4.0
Sudden Awakening from Sleep with Gasping & Choking	7	6.9
Awakening with Dry Mouth/Sore Throat	33	32.7
Morning Headache	12	11.9
Difficulty Concentrating During Daytime	14	13.9
Mood Changes	18	17.8
Night Sweat	4	4.0
Decreased Sex Drive	7	6.9

**Table 2:** Frequency distribution of medical comorbidities with OSA symptoms.

	**Excessive Daytime Sleepiness**	**Loud Snoring**	**Stopped Breathing during Sleep**	**Sudden Awakening from Sleep with Gasping & Choking**	**Awakening with Dry Mouth/Sore Throat**
Hypertension	9 (56.3%)	7 (43.8%)	1 (6.3%)	1 (6.3%)	5 (31.3%)
High Cholesterol	5 (50.0%)	4 (40.0%)	1 (10.0%)	0	2 (20.0%)
Heart Disease	1 (50.0%)	2 (100.0%)	0	1 (50.0%)	2 (100.0%)
Diabetes	4 (66.7%)	2 (33.3%)	1 (16.7%)	0	3 (50.0%)
Asthma	2 (33.3%)	1 (16.7%)	0	1 (16.7%)	0
GERD	1 (50.0%)	1 (50.0%)	0	0	1 (50.0%)
Cancer	2 (50.0%)	1 (25.0%)	0	0	3 (75.0%)
Depression	2 (66.7%)	1 (33.3%)	0	1 (33.3%)	3 (100.0%)
Deviated Nasal Septum	2 (66.7%)	1 (33.3%)	1 (33.3%)	1 (33.3%)	3 (100.0%)
Tonsillitis	3 (50.0%)	1 (16.7%)	0	1 (16.7%)	4 (66.7%)
Allergic Rhinitis	2 (22.2%)	1 (11.1%)	0	2 (22.2%)	2 (22.2%)
Obesity	3 (33.3%)	5 (55.6%)	0	0	2 (22.2%)
	**Morning Headache**	**Difficulty Concentrating during Daytime**	**Mood Changes**	**Night Sweat**	**Decreased Sex Drive**
Hypertension	2 (12.5%)	2 (12.5%)	2 (12.5%)	1 (6.3%)	1 (6.3%)
High Cholesterol	1 (10.0%)	2 (20.0%)	2 (20.0%)	1 (10.0%)	1 (10.0%)
Heart Disease	0	0	0	0	0
Diabetes	1 (16.7%)	2 (33.3%)	1 (16.7%)	1 (16.7%)	0
Asthma	0	0	0	0	0
GERD	0	0	0	0	0
Cancer	0	1 (25.0%)	1 (25.0%)	1 (25.0%)	0
Depression	1 (33.3%)	2 (66.7%)	3 (100%)	1 (33.3%)	1 (33.3%)
Deviated Nasal Septum	3 (100.0%)	2 (66.7%)	1 (33.3%)	0	0
Tonsillitis	3 (50.0%)	3 (50.0%)	3 (50.0%)	0	1 (16.7%)
Allergic Rhinitis	3 (33.3%)	3 (33.3%)	3 (33.3%)	0	1 (11.1%)
Obesity	1 (11.1%)	2 (22.2%)	2 (22.2%)	1 (11.1%)	1 (11.1%)

Abbreviations: GERD: Gastroesophageal Reflux Disease.

**Table 3:** Descriptive statistics of upper airway dimension as measured by CBCT imaging.

	N	Minimum	Maximum	Mean	Std. Deviation
Length (mm)	101	29.93	55.75	42.63	6.24
Total volume (cm^3^)	101	94.58	173.45	131.67	17.91
Average volume (cm^3^)	101	3.34	32.09	11.10	5.21
Antero-posterior (mm)	101	1.00	14.50	6.44	3.19
Width (mm)	101	8.00	37.00	21.69	6.54
Valid N (listwise)	101				

**Table 4:** Logistic regression analysis between medical comorbidities and upper airway dimensions.

		Length (mm)	Total Volume (cm^3^)	Average Volume (cm^3^)	Anterior-Posterior (mm)	Width (mm)
Hypertension	Odds Ratio	1.341	0.896	1.158	- *	0.781
	95% CI	1.056–1.705	0.819–0.980	0.893–1.503	- *	0.648–0.942
	*p*-value	0.016	0.017	0.269	- *	0.010
High Cholesterol	Odds Ratio	1.077	0.965	1.077	1.022	0.960
	95% CI	0.848–1.367	0.891–1.046	0.798–1.453	0.744–1.403	0.799–1.155
	*p*-value	0.544	0.383	0.628	0.894	0.668
Heart Disease	Odds Ratio	2.083	0.728	1.300	0.971	0.837
	95% CI	0.858–5.057	0.519–1.020	0.539–3.136	0.338–2.786	0.515–1.361
	*p*-value	0.105	0.065	0.559	0.956	0.473
Diabetes	Odds Ratio	1.163	0.919	1.177	0.918	0.845
	95% CI	0.834–1.622	0.816–1.035	0.762–1.815	0.595–1.415	0.659–1.085
	*p*-value	0.373	0.165	0.463	0.697	0.186
Asthma	Odds Ratio	0.841	1.047	1.297	0.941	0.770
	95% CI	0.619–1.142	0.951–1.153	0.856–1.964	0.632–1.402	0.585–1.014
	*p*-value	0.267	0.347	0.219	0.766	0.062
GERD	Odds Ratio	22.034	0.271	0.000	0.000	0.766
	95% CI	0.000–2.584	0.000–1.020	0.000	0.000	0.000
	*p*-value	0.990	0.992	0.993	0.982	1.000
Cancer	Odds Ratio	1.694	0.822	1.413	0.735	0.778
	95% CI	0.966–2.971	0.671–1.006	0.789–2.528	0.403–1.341	0.540–1.122
	*p*-value	0.066	0.058	0.245	0.316	0.179
Depression	Odds Ratio	1.053	0.993	0.693	1.352	1.124
	95% CI	0.693–1.599	0.870–1.133	0.342–1.402	0.770–2.372	0.795–1.589
	*p*-value	0.810	0.918	0.308	0.294	0.507
Deviated Nasal Septum	Odds Ratio	1.039	0.992	0.637	1.502	1.189
	95% CI	0.674–1.599	0.865–1.139	0.310–1.307	0.847–2.663	0.831–1.702
	*p*-value	0.864	0.914	0.219	0.164	0.344
Tonsillitis	Odds Ratio	1.124	1.020	0.671	1.190	1.023
	95% CI	0.829–1.523	0.932–1.115	0.365–1.231	0.750–1.889	0.770–1.358
	*p*-value	0.451	0.668	0.197	0.461	0.876
Allergic Rhinitis	Odds Ratio	0.851	1.029	0.846	1.199	1.112
	95% CI	0.655–1.106	0.951–1.113	0.577–1.241	0.846–1.698	0.903–1.368
	*p*-value	0.227	0.477	0.392	0.308	0.318
Obesity	Odds Ratio	1.175	0.953	1.042	1.086	0.792
	95% CI	0.876–1.577	0.864–1.051	0.687–1.581	0.745–1.583	0.619–1.014
	*p*-value	0.282	0.333	0.845	0.669	0.899

* Linearity of independent variables and log odds not achieved; hence, results obtained are not valid. Significant associations: *p* ≤ 0.05 and if 1 does not fall between lower bound and upper bound of CI are bold. Abbreviations: CI: Confidence Interval; GERD: Gastroesophageal Reflux Disease.

**Table 5:** Logistic regression analysis between OSA symptoms and upper airway dimensions.

		Length (mm)	Total Volume (cm^3^)	Average Volume (cm^3^)	Anterior-Posterior (mm)	Width (mm)
Excessive Daytime Sleepiness	Odds Ratio	1.159	0.979	0.843	1.243	1.010
	95% CI	0.998–1.345	0.934–1.026	0.679–1.045	1.008–1.533	0.893–1.142
	*p*-value	0.053	0.373	0.118	0.042	0.877
Loud Snoring	Odds Ratio	1.154	- *	0.711	0.908	1.052
	95% CI	1.022–1.304	- *	0.515–0.980	0.692–1.191	0.897–1.233
	*p*-value	0.021	- *	0.037	0.484	0.532
Observed episodes of breathing stopping during sleep	Odds Ratio	1.118	0.997	0.626	1.018	1.185
	95% CI	0.806–1.551	0.901–1.103	0.319–1.231	0.582–1.780	0.887–1.584
	*p*-value	0.504	0.955	0.175	0.950	0.251
Abrupt awakening from sleep accompanied by gasping or choking	Odds Ratio	0.985	1.053	0.840	1.057	1.111
	95% CI	0.764–1.269	0.976	0.557–1.267	0.723–1.545	0.876–1.409
	*p*-value	0.906	1.137	0.406	0.775	0.386
Awakening with a dry mouth or sore throat	Odds Ratio	1.119	0.982	0.928	- *	1.029
	95% CI	0.976–1.283	0.937–1.030	0.791–1.089	- *	0.917–1.155
	*p*-value	0.107	0.458	0.362	- *	0.627
Morning headache	Odds Ratio	1.108	0.988	0.796	1.333	1.118
	95% CI	0.889–1.382	0.921–1.059	0.579–1.095	0.971–1.828	0.929–1.344
	*p*-value	0.361	0.733	0.161	0.075	0.237
Difficulty concentrating during the day	Odds Ratio	1.275	1.014	0.471	2.097	1.252
	95% CI	0.994–1.637	0.945–1.089	0.291–0.765	1.358–3.238	0.995–1.576
	*p*-value	0.056	0.696	0.002	<0.001	0.056
Mood changes	Odds Ratio	1.101	1.015	0.729	1.477	1.133
	95% CI	0.912–1.330	0.958–1.075	0.542–0.980	1.103–1.979	0.960–1.339
	*p*-value	0.316	0.619	0.036	0.009	0.141
Night-time sweating	Odds Ratio	1.421	0.916	0.533	1.599	1.160
	95% CI	0.872–2.316	0.786–1.067	0.245–1.159	0.871–2.937	0.847–1.588
	*p*-value	0.158	0.260	0.112	0.130	0.356
Decreased sex drive	Odds Ratio	1.143	0.964	0.647	1.500	1.144
	95% CI	0.841–1.552	0.873–1.064	0.392–1.066	0.995–2.261	0.899–1.457
	*p*-value	0.393	0.466	0.087	0.053	0.274

* Linearity of independent variables and log odds not achieved; hence, the results obtained are not valid. Significant associations: *p* ≤ 0.05 and if 1 does not fall between lower bound and upper bound of CI are bold.

**Table 6:** Fisher’s exact test (*p*-value) between medical comorbidities and OSA symptoms.

	**Excessive Daytime Sleepiness**	**Loud Snoring**	**Stopped Breathing During Sleep**	**Sudden Awakening from Sleep with Gasping & Choking**	**Awakening with Dry Mouth/Sore Throat**
Hypertension	0.093	0.016	0.504	1.000	1.000
High Cholesterol	0.491	0.106	0.345	1.000	0.492
Heart Disease	1.000	0.038	1.000	0.134	0.105
Diabetes	0.188	0.340	0.220	1.000	0.389
Asthma	1.000	1.000	1.000	0.358	0.174
GERD	1.000	0.358	1.000	1.000	0.549
Cancer	0.622	1.000	1.000	1.000	0.101
Depression	0.552	0.488	1.000	0.196	0.033
Deviated Nasal Septum	0.552	0.488	0.115	0.196	0.033
Tonsillitis	0.666	1.000	1.000	0.358	0.087
Allergic Rhinitis	0.480	0.684	1.000	0.118	0.714
Obesity	1.000	0.014	1.000	1.000	0.714
	**Morning Headache**	**Difficulty Concentrating during Daytime**	**Mood Changes**	**Night Sweat**	**Decreased Sex Drive**
Hypertension	1.000	1.000	0.730	0.504	1.000
High Cholesterol	1.000	0.626	1.000	0.345	0.529
Heart Disease	1.000	1.000	1.000	1.000	1.000
Diabetes	0.541	0.193	1.000	0.220	1.000
Asthma	1.000	0.592	0.588	1.000	1.000
GERD	1.000	1.000	1.000	1.000	1.000
Cancer	1.000	0.455	0.550	0.151	1.000
Depression	0.319	0.050	0.005	0.115	0.196
Deviated Nasal Septum	0.001	0.050	0.449	1.000	1.000
Tonsillitis	0.021	0.034	0.068	1.000	0.358
Allergic Rhinitis	0.072	0.108	0.198	1.000	0.491
Obesity	1.000	0.608	0.660	0.316	0.491

Significant associations (*p* ≤ 0.05) are bold. Abbreviations: GERD: Gastroesophageal Reflux Disease.

## Data Availability

Data will be made available upon request to experts in the field.
